# An expression meta-analysis of predicted microRNA targets identifies a diagnostic signature for lung cancer

**DOI:** 10.1186/1755-8794-1-61

**Published:** 2008-12-16

**Authors:** Yu Liang

**Affiliations:** 1 Division of Molecular Cell Biology-Assay R&D, Applied Biosystems, 850 Lincoln Centre Drive, Foster City, CA 94404, USA

## Abstract

**Background:**

Patients diagnosed with lung adenocarcinoma (AD) and squamous cell carcinoma (SCC), two major histologic subtypes of lung cancer, currently receive similar standard treatments, but resistance to adjuvant chemotherapy is prevalent. Identification of differentially expressed genes marking AD and SCC may prove to be of diagnostic value and help unravel molecular basis of their histogenesis and biologies, and deliver more effective and specific systemic therapy.

**Methods:**

MiRNA target genes were predicted by union of miRanda, TargetScan, and PicTar, followed by screening for matched gene symbols in NCBI human sequences and Gene Ontology (GO) terms using the PANTHER database that was also used for analyzing the significance of biological processes and pathways within each ontology term. Microarray data were extracted from Gene Expression Omnibus repository, and tumor subtype prediction by gene expression used Prediction Analysis of Microarrays.

**Results:**

Computationally predicted target genes of three microRNAs, miR-34b/34c/449, that were detected in human lung, testis, and fallopian tubes but not in other normal tissues, were filtered by representation of GO terms and their ability to classify lung cancer subtypes, followed by a meta-analysis of microarray data to classify AD and SCC. Expression of a minimal set of 17 predicted miR-34b/34c/449 target genes derived from the developmental process GO category was identified from a training set to classify 41 AD and 17 SCC, and correctly predicted in average 87% of 354 AD and 82% of 282 SCC specimens from total 9 independent published datasets. The accuracy of prediction still remains comparable when classifying 103 AD and 79 SCC samples from another 4 published datasets that have only 14 to 16 of the 17 genes available for prediction (84% and 85% for AD and SCC, respectively). Expression of this signature in two published datasets of epithelial cells obtained at bronchoscopy from cigarette smokers, if combined with cytopathology of the cells, yielded 89–90% sensitivity of lung cancer detection and 87–90% negative predictive value to non-cancer patients.

**Conclusion:**

This study focuses on predicted targets of three lung-enriched miRNAs, compares their expression patterns in lung cancer by their GO terms, and identifies a minimal set of genes differentially expressed in AD and SCC, followed by validating this gene signature in multiple published datasets. Expression of this gene signature in bronchial epithelial cells of cigarette smokers also has a great sensitivity to predict the patients having lung cancer if combined with cytopathology of the cells.

## Background

Lung cancer is the most common cause of cancer mortality in the world [1], and over 50% of the cases are diagnosed as AD and SCC. These two subtypes present unique histopathological characteristics at distinctive preferential anatomic locations, and yet are classified together as non-small cell lung cancer (NSCLC) and their staging and treatment are similar. Standard treatment strategies include surgical resection, followed by radiation and/or chemotherapy. Chemotherapy, especially for advance-stage disease, is usually palliative rather than curative due to resistance [2], so more effective systemic therapy is in urgent need. One solution might be developing novel agents to target specific pathways in different tumor subtypes [3]. Gene expression signatures that can characterize the heterogeneity of lung cancer would provide molecular underpinnings of the histopathological features and the regulatory networks for tumor subtypes and lead to identification of molecular targets for such agents [4].

Late diagnosis of extensive diseases is the main reason for treatment failure in lung cancer. Although a recent large randomized study suggested that computed tomography screening may not decrease number of diagnosis of advanced lung cancer and death from the tumor [5], early detection may still be a key to improve survival of early-stage patients, particularly if more sensitive and specific diagnostic tools can be developed. Abnormal radiographic imaging requires further examination such as bronchoscopy or biopsy, but these procedures are highly variable in diagnostic yield and not very sensitive to small peripheral tumors (only 30~40% in accuracy) [6]. It will be cost efficient for clinical management if evaluation of specific gene signature in specimens taken by bronchoscopy could eliminate false-positive cases to reduce subsequent unnecessary tests and treatments [7].

MicroRNAs (miRNAs) are a group of small non-protein coding RNAs that often demonstrate temporally or spatially restricted expression patterns, and have great potential to mark the cytological and histological origins of tissues with less clear differentiated phenotypes, such as poorly differentiated tumors [8]. In our previous report, expression of 345 miRNAs was profiled in 40 normal human tissue types, providing a reference in abundance for identification of tissue-specific or tissue-enriched miRNAs [9]. It is believed that miRNAs post-transcriptionally regulate gene expression through at least two mechanisms: translational inhibition or degradation of mRNAs. Therefore, functional roles of a miRNA should be determined by its target genes. It has been shown that many tissue-specific factors have reduced expression in cancers derived from the tissues in which these factors are specifically expressed [10,11]. For tissue-specific miRNAs that are under-expressed in the neoplastic tissue counterparts, deregulation of their target genes in these disease lesions would likely characterize the pathobiology of the tumor.

Current identification of miRNA targets mainly follows computational prediction, and only a handful of them have been experimentally verified. Many investigations presumed expression of miRNA is opposite to that of their targets, based on observations in which transfecting miRNAs into human cells down-regulated large number of target mRNAs [12]. A major drawback of this strategy is its lack of considering target genes whose protein but not transcript levels are down regulated by the miRNAs. In fact, a recent report using biochemical purification showed that, among the genes co-immunoprecipitated with the RNA-induced silencing complex after the miRNA transfection, about 70% of them have no change in mRNA abundance but their 3'-end untranslated regions actually mediate reduced protein expression of a reporter gene in response to transfection of the same miRNA [13].

In this study, expression of all predicted target genes of three lung-enriched miRNAs was examined in different subtypes of lung cancer without relating to matched miRNA expression. After a series of data filtering by classification of tumor subtypes, a minimal gene signature was identified to correctly predict the majority of AD and SCC cases from multiple published datasets. Expression of the same signature was also examined in two airway epithelial cell gene expression datasets to evaluate its diagnostic value for early detection of lung cancer in cigarette smokers.

## Methods

### Prediction and filtering of miRNA target genes

In general, the union of combining different prediction algorithms gives much greater number of predicted miRNA target genes, and therefore less false negatives and more false positives, than individual algorithms or the intersection of combining different algorithms. The strategy used in this study was to obtain maximally possible number of candidates, followed by a series of data reduction to filter out genes. MiRNA target genes were predicted by union of miRBase Target v4 (powered by miRanda), TargetScan 4.0, and PicTar, followed by screening for availability of gene symbols in NCBI human sequences and Gene Ontology (GO) terms using the PANTHER database v6 [14]. Genes with eligible symbols available and being categorized by GO terms were retained. The number of genes in each category within the Biological Processes Ontology term and representation of each category were examined: developmental processes, nucleotide metabolism, and signal transduction are the top three categories with the largest number of genes and the smallest corrected p values (see below). PANTHER was also used for analyzing the significance of biological processes and pathways within each ontology term.

### Extraction of microarray data from public databases

**Figure 1:**
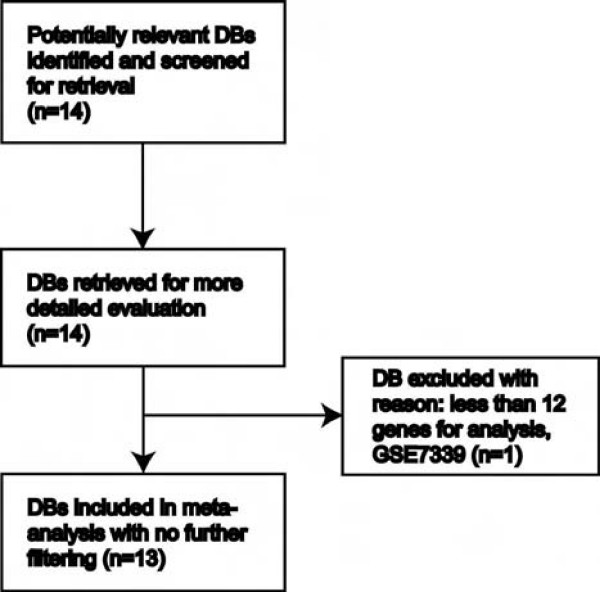
**A flow diagram outlines selection of 13 human lung cancer databases used in this study**. This diagram does not include the 14^th ^dataset GSE2514 in Table 1 that is a mouse gene expression dataset and was not used to calculate the accuracy of prediction in the meta-analysis. DB, database.

Microarray data were extracted from Gene Expression Omnibus repository, and all databases used in this study were summarized in Table 1. To comply with the Consolidated Standards of Reporting Trials statement [15], selection of databases using human specimens investigated in this study and the key findings are summarized in the Additional file 1 and the flow diagram for the rationale of database selection is summarized in Figure 1. All microarray datasets from Gene Expression Omnibus using human primary lung cancer specimens (no cell lines) under the search terms "human lung adenocarcinoma" or "human lung squamous carcinoma" as of July 1^st^, 2007 were reviewed. Only one dataset, GSE7339, was removed from further analysis due to too many genes missing from the 17-gene signature for prediction analysis. The 14^th ^dataset GSE2514 in Table 1 that is a mouse gene expression dataset was not applied to the diagram in Figure 1 and was not included in calculating the accuracy of prediction in the meta-analysis. In studies containing multiple probes from a given gene symbol, one was randomly selected and subject to analyses.

**Table 1:** Summary of lung cancer datasets used in this study.

Database	GEO	Platform	Institute	PMID	Technology type	Organism	AD#	SCC#	Stage	# Genes	% Correct AD	% Correct SCC
1	GSE3398	GPL2648/2778/2832	Stanford	11707590	spotted cDNA	Human	41	17	I to III	17	93	94
2	NA*	Affy HG-U95A	DFCI	11707567	oligonucleotide	Human	139	21	I to III	17	91	86
3	NA**	Affy HG-U133A	U Michigan	12118244	oligonucleotide	Human	86	0	I to III	17	95	NA
4	GSE3141	Affy U95A/HuGeneFL	Duke	16899777	oligonucleotide	Human	54	57	I to III	17	83	72
5	GSE4573	Affy HG-U133A	U Michigan	16885343	oligonucleotide	Human	0	129	I to III	17	NA	85
6	GSE1037	CHUGAI 41K	CIH, Japan	15016488	spotted cDNA	Human	12	0	NA	17	83	NA
7	GSE6253	Affy HG-U95A/U133AB	Washington U	17194181	oligonucleotide	Human	14	36	I	17	79	78
8	GSE3268	Affy HG-U133A	UC Davis	16188928	oligonucleotide	Human	0	5	NA	17	NA	100
9	GSE1987	Affy HG-U95A	Tel Aviv U	NA	oligonucleotide	Human	8	17	I to III	17	88	59
10	GSE6044	Affy HG-Focus	U Duesseldorf, Germany	NA	oligonucleotide	Human	10	10	NA	16	70	90
11	GSE7880	Affy HG-Focus	Heinrich-Heine U, Germany	NA	oligonucleotide	Human	25	18	IIIB/IV	16	92	83
12	GSE2514	Affy HG-U95A	U Colorado	16314486	oligonucleotide	Human	20	0	NA	15	100	NA
13	GSE5843 GSE5123	PC Human Operon v2	Prince Charles H, Australia	17082175 17504995	oligonucleotide	Human	48	51	I to III	14	75	80
14	GSE2514	Affy MG-U74A	U Colorado	16314486	oligonucleotide	Mouse	44	0	NA	13	100	NA

*

**

### Clustering analysis and evaluation of classification of lung cancer with miRNA target genes

Clustering of gene expression profiles used Gene Cluster 3.0, and the parameters used are the following: after removing genes that had good data in less than 80% of specimens examined, data were adjusted by gene centering only (no array centering), and the hierarchical clustering used uncentered correlation as the similarity metric and average linkage as the clustering method. Classification of lung cancer specimens by miRNA target genes was compared to the clustering profiles of mRNA expression in the original article publishing the Database 1 [16]. Predicted target genes were categorized by biological process ontology terms, and the one among the 4 categories with the largest number of genes that has the most similar clustering patterns to the mRNA expression profiles of the specimens was chosen for the next step of class prediction; AD and SCC subtypes were used for subsequent classification by the gene expression signature because they are the best classified by these genes among all the histologic subtypes examined. For the detail why AD and SCC were selected but not small cell lung carcinoma (SCLC) and large cell lung carcinoma (LCLC), see below and Additional file 2 for detail.

### Data adjustment and prediction of tumor subtypes

Because microarray datasets use different platforms and the data are presented in different formats, the microarray data were adjusted to similar scale and distribution patterns before class prediction based on four guidelines (for detail, see Additional file 2). First, for microarrays spotted with cDNA clones presented with log2-transformed data, no adjustment was made. Second, for data from oligonucleotide-arrays (for example, Affymetrix) that has not been log2-transformed, ratio of each data point to the average expression of the 17 "core" genes across all specimens was calculated, followed by log3-transformation, because log3-transformed data have a better overlapped distribution with the cDNA-spotted microarray data compared to log2 transformation. To be consistent, data from all oligonucleotide arrays were log3-transformed with a few exceptions. For detail, see Additional file 2. Third, for data from oligonucleotide-arrays that has been log2-transformed, each data point was raised to the power of 2 and re-log3-transformed. Fourth, for data from oligonucleotide-arrays in which their log-base is unknown or data formats are unusual and therefore cannot be justified using data distribution, a simple ratio between each data point and the average expression was computed. Gene expression was trained using Prediction Analysis of Microarrays (PAM) Version 2.13 using the nearest shrunken centroid algorithm [17]. Missing microarray data were handled by the k-nearest neighbor imputation engine using 10 nearest neighbors. When the 17-gene signature was identified, expression data of these 17 genes (or any number of genes that are available in the selected datasets) were extracted. In the subsequent prediction analysis, the Database 1 continues to serve as the training set, and each one of the rest of datasets was the test set for prediction. Since all 17 genes were used, the threshold was set to 0 (used all genes) and follows the standard procedure of PAM for the rest of steps without any modification from the instruction. Significance Analysis of Microarrays (SAM) was used to demonstrate the false detection rate for separating AD from SCC in the Database 1 by the 17-gene signature.

## Results

### Independent studies demonstrated reduced expression of miR-34b/34c in lung cancer

**Figure 2:**
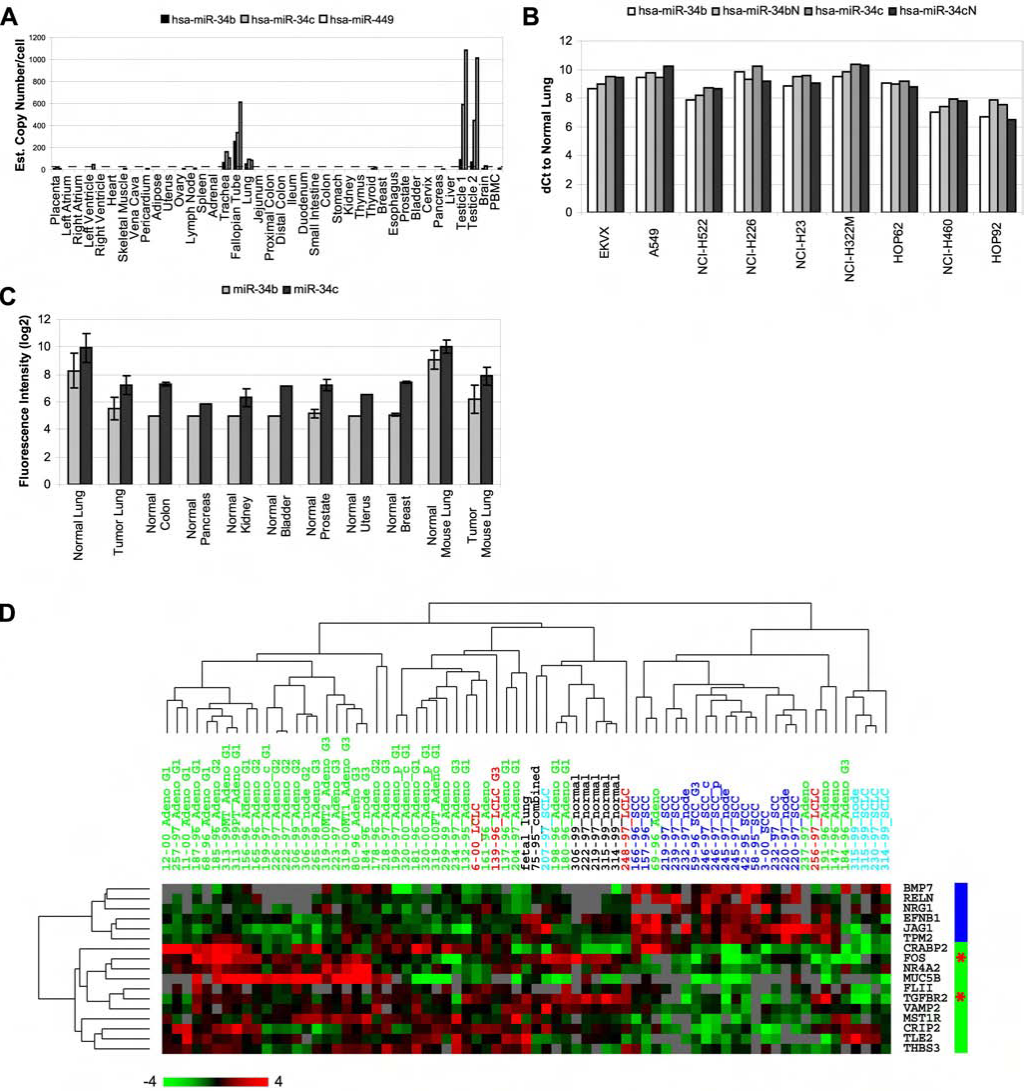
***A*****, expression of miR-34b/34c/449 is enriched in normal lung, fallopian tube, and testis**. C_T _higher than 35 is generally considered as less reliable, and equals to around 5 copies of miRNA. Dashed line: around 20 copies of miRNA. Expression of these 3 miRNAs is much higher than this line in lung, fallopian tube, and testis than in the rest of tissues examined. *B*, expression of miR-34b/34c/449 is reduced in the 9 lung cancer cell lines of the NCI-60 panel compared to normal lung, represented by ΔC_T _(tumor cell line – normal lung). *C*, expression of miR-34b/34c is reduced in both human and mouse primary lung cancer tissues compared to normal lung. The log2-based background fluorescence intensity is between 5 and 6, so tumor lung and non-lung normal human tissues have essentially no miR-34b expression and some minimally detectable miR-34c, consistent with the TaqMan^®^-based results in the panel *A*. *D*, expression of the 17 "core" genes in specimens from the Database 1; blue bar, genes with higher expression in SCC; red bar, genes with higher expression in AD; *, two genes with higher expression in normal lung; scale bar represents fold change while gray in the heat map indicates missing data.

A search of miRNAs preferentially expressed in normal lung in our previously published dataset found a group of 3 miRNAs (miR-34b, miR-34c, and miR-449) that had approximately a thousand copies or less each cell in testes, fallopian tubes, lung, and trachea, while the rest of tissues examined had no or barely detectable levels of expression [9] (Figure 2A). Lung-enriched expression of miR-34b/34c was also observed in three other independent studies. In one report expression of miR-34b and 34c were higher than 7 other tissue types [8], while the other one showed that precursor miR-34b and 34c expression was primarily in lung and testis [18]. The third report did not have information for miR-34c but showed increased expression of miR-34b in lung [19]. The promoter regions of miR-34b/34c genes have potential p53-binding sites that have been experimentally verified, and both miRNAs are part of the p53 tumor suppressor network [20]. Although miR-449 lacks such information as transcriptional regulation and functional roles, a web-based tool [21] that identifies potential *cis*-regulatory elements by comparative genomics recognizes a p53-binding site within a region about 1.5 kb upstream to the miR-449 gene (see Additional file 2 and its Figure 1).

**Figure 3:**
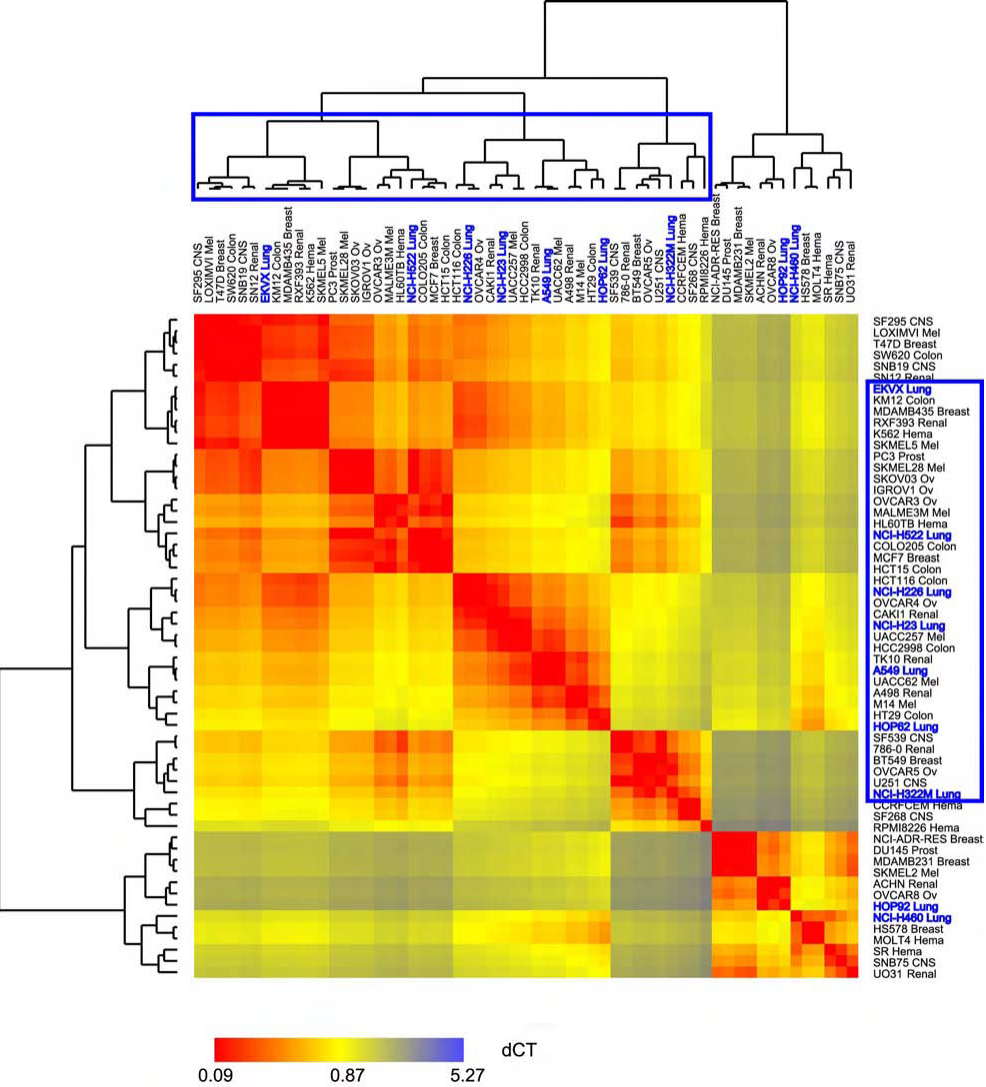
**A similarity metric demonstrates the correlation of miR-34b/34c expression between lung cancer cell lines (blue letters)**. All NSCLC cell lines are in the blue bracket, suggesting that expression of miR-34b/34c/449 might be candidates to classify different types of lung cancer. The calculation of the distance matrix was based on the ΔC_T _(the average C_T _of miR-34b/34bN/34c/34cN between two cell lines), so the smaller the ΔC_T_, the more similar expression levels of the four miRNA sequences are in the two cell lines (more red in the heat map).

Two independent datasets were used to test the hypothesis whether these miRNAs (miR-449 was not available in these two databases) had reduced expression in lung cancer. One is from our previous published miRNA expression profiles in the NCI-60 panel of cell lines derived from human cancers that used real-time PCR for quantitation [22], and the expression of miR-34b and miR-34c in 9 cell lines derived from lung was compared with that in normal lung tissue obtained from our body map data [9] (Figure 3, and Addition file 2 and its Table 1). Expression of the four miRNA sequences quantitated in this study (miR-34b/34bN and miR-34c/34cN) in normal lung is from 90-fold to over 1,300-fold higher than in any of the lung cancer cell lines tested (Figure 2B). Another dataset used a bead-based technology to quantitate miRNAs [8], and the expression of both miR-34b and miR-34c is again significantly higher in normal lung than in 6 lung tumor specimens (Figure 2C, p = 0.004 and 0.002, respectively, by t-test). The same significant difference was observed between normal mouse lung and tumor specimens from lung tissues of mice carrying oncogenic K-ras [23] (Figure 2C). This echoes the observation made by a separate group in which miR-34b expression was found decreased by more than 90% in 4 out of 5 AD and 2 out of 8 SCC [19].

### Predicted target genes of miR-34b/34c/449 ontologically termed with developmental processes distinguish lung adenocarcinomas from squamous cell carcinomas

It has been shown that the union of miRNA target genes predicted by three computational algorithms (miRanda, PicTar, and TargetScan) is one of the strategies that give the highest sensitivity [24], which predicted total 2414 unique gene symbols targeted by miR-34b/34c/449 (see Additional file 3 for complete list of genes), and 2033 of these genes were categorized in GO. The 4 largest categories in the biological process ontology term are signal transduction (361 genes, 17.8%), nucleoside, nucleotide, and nucleic acid metabolism (358 genes, 17.6%), and developmental processes (251 genes, 12.3%). Protein metabolism and modification (300 genes, 14.8%) constitutes the third largest group but has a much higher p value than the other 3 groups and therefore was excluded from further analysis (see Additional File 2 and Additional file 4). Using the Stanford lung cancer dataset (Database 1 in Table 1) [16] that contains four major subtypes of lung cancer (AD, SCC, SCLC, and LCLC), unsupervised classification of the specimens with genes from these three GO categories was compared with the classification profiles using the 918 cDNA clones selected by the authors in the original article. This result suggests that the developmental process genes might better distinguish AD from SCC, but not as well to classify SCLC and LCLC (see Additional file 2 and its Figure 2). Using AD, SCLC, and LCLC specimens from Database 6 [25] to train the 251 developmental process genes for predicting the four tumor subtypes in Database 1 further supports the use of these genes to classify AD and SCC (see Additional file 2 and its Figure 3).

### Expression of a 17-gene signature correctly predicts most lung adenocarcinomas and squamous cell carcinomas in independent datasets

The developmental process genes with eligible data points in the Database 1 were re-analyzed by PAM as a training set. After cross-validation, the error plot shows the lowest error rate between threshold 0 and 1.6, indicating that the genes represented by the threshold 1.6 is the minimal subset of "core genes" that can best separate the 41 AD and 17 SCC in this dataset (which outputs the 17 genes). SAM algorithm also showed that these 17 genes have the lowest false detection rate to separate AD from SCC (q value equals to 0). Examining the predicted target sites of miR-34b/34c/449 in these 17 genes showed that most of the genes have target sites predicted by at least 2 of the 3 algorithms and at least 2 target sites predicted by at least one of the algorithms (Table 2). Six of these 17 genes have increased expression in all SCC, while 9 genes have higher expression in at least a subset of AD than in normal lung (Figure 2D). Expression of the other two genes, FOS and TGFBR2, is reduced in tumor tissues, which is in agreement with the literature [26-28]. Five out of the 17 genes (CRABP2, CRIP2, FOS, JAG1, and MST1R) were among the differentially expressed markers between AD and SCC in the original report publishing the Database 1 [16]. Two genes, BMP7 and JAG1, were also among the 23 genes that had 4-fold or greater expression in SCC than in AD reported in a separate paper [29].

**Table 2:** The frequency of predicted miR-34b/34c/449 target sites within the 17 core genes

Algorithm	TargetScan 4.0				MiRanda v4			PicTar	
miRNA	34b (c*)	34b (pc**)	34/449 (c*)	34/449 (pc**)	34b	34c	449	34b	34c
									
BMP7		1			1				
CRABP2^§^				1		2	1		
CRIP2^§^				1		1	1		
EFNB1		1	1	2		1	1		
FLII					1	1			
FOS^§^				1		1			
JAG1^§^	1		1	1			1	1	1
MST1R^§^					1				
MUC5B						1	2		
NR4A2	1	1						2	2
NRG1					1			3	3
RELN			1		1				
TGFBR2		2		1	1	1			
THBS3						2	2		
TLE2		1					1		
TPM2							2		
VAMP2	1	1	3					1	1

*c: Conserved sites for miRNA families conserved in human, mouse, rat, and dog.

**pc: Poorly conserved sites and sites for poorly conserved miRNA families.

§Genes also reported as differentially expressed between AD and SCC in the paper that published the Database 1.

**Figure 4:**
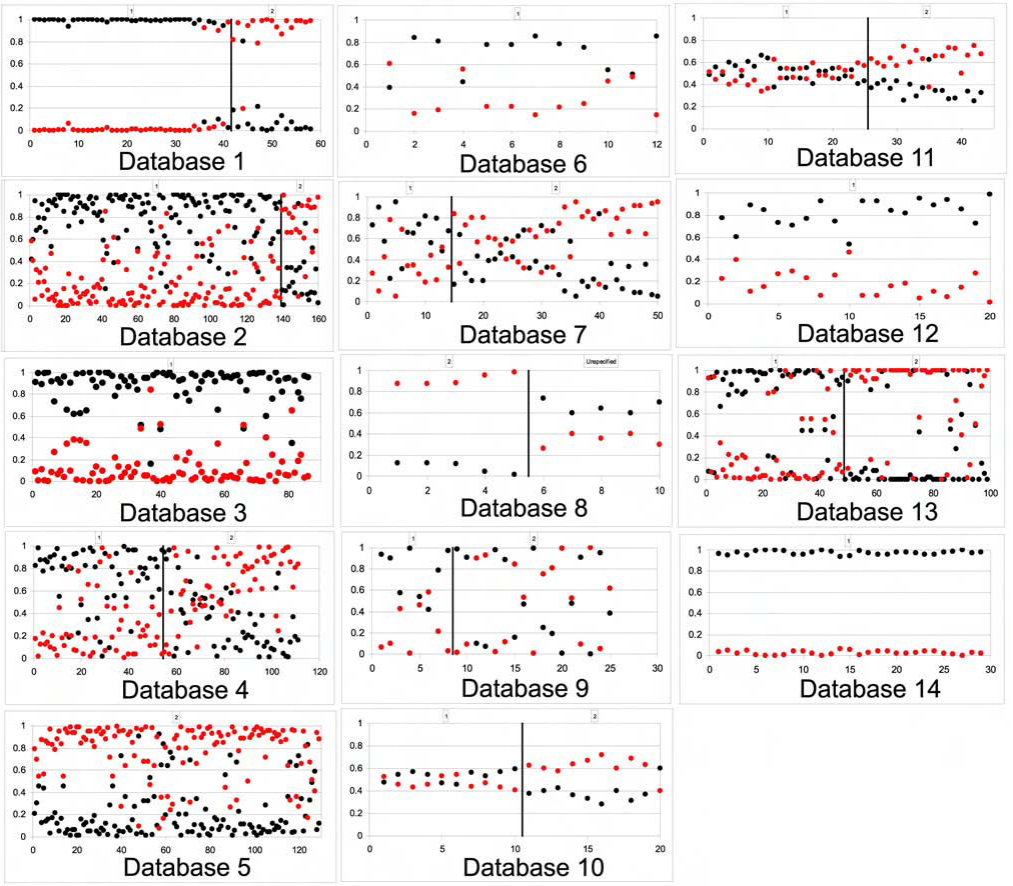
**The test probabilities (Y-axis) of predicting AD and SCC specimens from 14 published datasets using the 17 "core" genes**. Group 1, AD; group 2, SCC; black circle, predicted as AD; red circle, predicted as SCC.

Expression of the 17 core genes in another 8 validation datasets [25,30-35] was used to predict the diagnosis of specimens as AD or SCC. There are in total 313 AC and 265 SCC specimens, and the expression of the 17 genes correctly predicted 79~100% of AD and 59~86% of SCC (Figure 4). There are another 4 datasets [36-38] that have fewer genes available for validation (14 to 16 genes, see Table 1) and have 103 AD and 79 SCC specimens in total, and still 70~100% of AD and 80~90% of SCC were correctly predicted (Figure 4).

The Database 14 contains 44 specimens derived from a urethane-induced mouse lung tumor model in which the neoplastic tissues exhibit histological appearance and molecular changes similar to human lung AD [38]. This dataset has only 13 of the 17 core genes available for analysis, and expression of these 13 genes can still correctly predict all specimens as AD. Together with the 4 datasets described above that had 14 to 16 of the 17 genes available, this result suggests that at least some datasets need only part of the 17 genes to distinguish AD from SCC, and therefore there might be signaling pathway(s) represented by genes from this signature underlies the unique biologies of these two subtypes.

### The transforming growth factor-beta pathway is overrepresented in the 17-gene signature

The pathway ontology terms of the 17 genes shows that only the TGF-beta signaling pathway is overrepresented after Bonferroni correction (3 genes, p = 0.02, see Additional file 2 and its Table 2). At the beginning of analysis, there were 29 genes out of the 2033 GO-termed genes that belong to the TGF-beta pathway (versus 11.96 genes expected, p = 0.011 by Fisher's exact test), and genes in this category was further enriched such that 16 out of the 251 developmental process genes (versus 1.57 genes expected, p = 0.00001 by Fisher's exact test) were in this category. Therefore, the emergence of TGF-beta signaling cascade does not appear to be a random event, but rather a specific enrichment to suggest its central role in AD and SCC.

**Figure 5:**
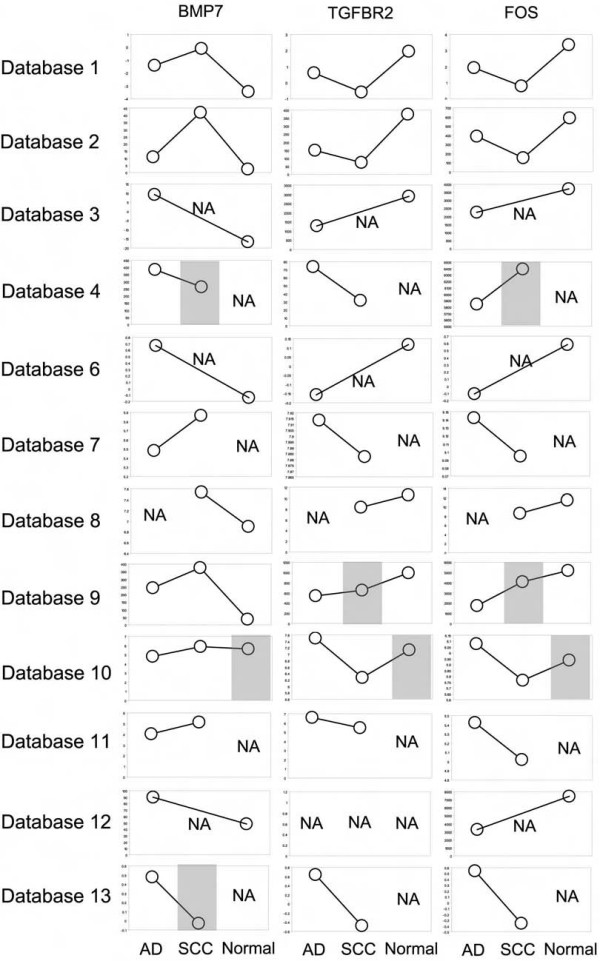
**Expression of BMP7, TGFBR2, and FOS was examined in AD, SCC, and normal lung from 12 of the 13 human gene expression databases**. Each circle represents the average expression of all specimens from the same tumor subtype using the original data format and scale. "NA", not available for analysis due to missing data. Gray shaded boxes indicate the relative expression levels of the genes are not coherent to the expected patterns.

Expression of BMP7, TGFBR2, and FOS was collectively compared in all datasets except for the Database 5 that had only SCC available. Among the 11 datasets, majority of them show that BMP7 is up-regulated and TGFBR2/FOS are down-regulated in both AD and SCC compared to normal lung, whereas between AD and SCC, BMP7 has an opposite expression pattern to TGFBR2/FOS (Figure 5). These results support that both AD and SCC subtypes have suppressed TGF-beta signaling but through different mechanisms: in SCC there is increased expression of BMP7 that antagonizes TGF-beta functions [39], and reduced expression of TGFBR2, which suggests that SCC might be more sensitive than AD to targeting BMP7 or increasing expression of TGFBR2. An additional analysis of another TGF-beta inhibitory molecule SMAD7 [40] that is not in the final 17-gene signature but in the 251 developmental process genes is also consistent with these results (see Additional file 2 and its Table 3).

Several randomized controls were used to compare with the process of identification of this 17-gene signature. Three miRNAs, miR-141/146b/216, were randomly chosen and all subsequently procedures followed the same workflow for the miR-34b/34c/449 including prediction of targets, screening of gene symbols and GO categories/terms, union of all selected genes, and clustering analysis of tumor specimens. Two random sets of 17 genes were also selected to use the Database 1 as the training set and predict AD/SCC in the Database 2. Classification and prediction of AD and SCC subtypes using the miR-34b/34c/449 prediction targets and the 17-gene signature, respectively, are clearly better than randomized controls. Detail of these analyses and selected genes are described in Additional files 2 and 5.

### Expression of the "core" gene signature has the potential to diagnose lung cancer using bronchoscopic specimens of cigarette smokers

**Figure 6:**
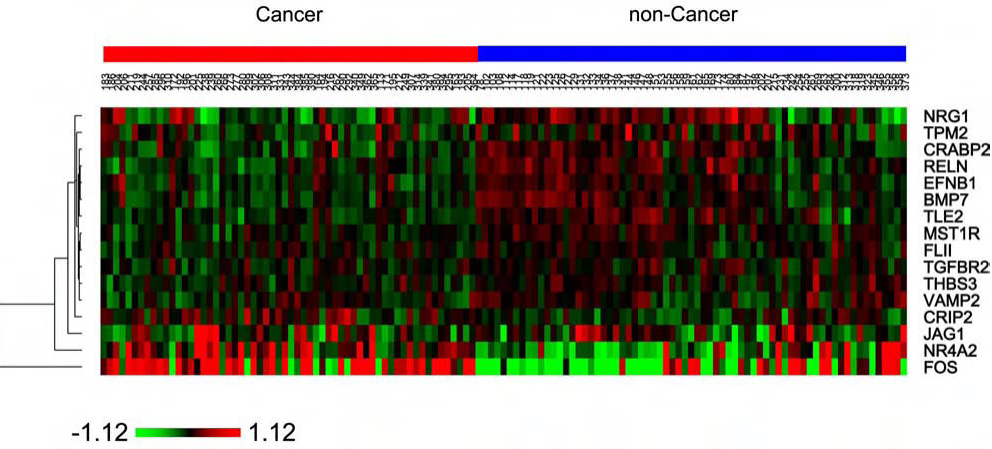
**Differential expression of the 16-gene signature in the specimens from cigarette smokers diagnosed with (N = 59, red bar) and without (N = 69, blue bar) lung cancer in the training set**. The scale bar represents the difference of Robust Multichip Average-generated expression value between each data point and the average expression value for each gene.

**Figure 7:**
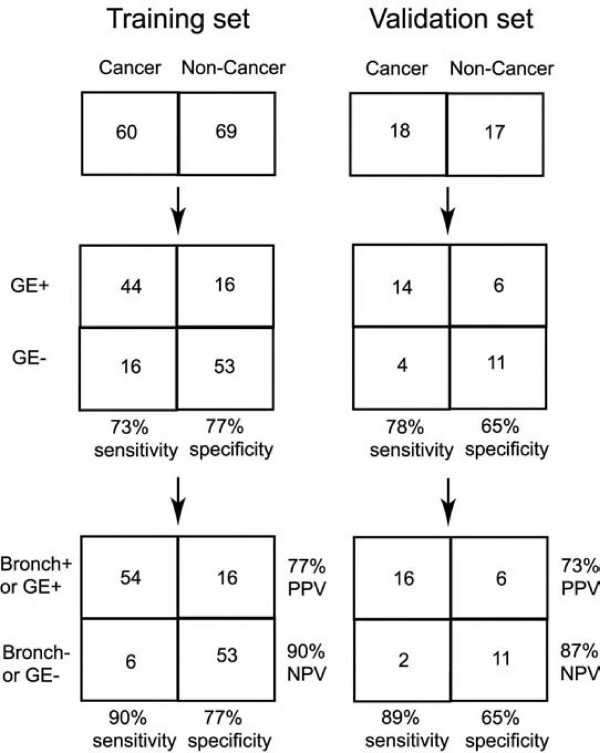
**Diagnostic evaluation of two microarray datasets of bronchoscopic specimens using expression of the 16-gene signature alone, and combination of gene expression and cytopathology**. GE+ and GE-, predicted as cancer and non-cancer by the signature, respectively. Bronch+ and Bronch-, cancer and non-cancer diagnosis by cytopathology examination. PPV and NPV, positive predictive value and negative predictive value, respectively.

The possibility of using this gene signature for early detection of lung cancer and predict tumor subtypes was evaluated using a published microarray dataset of large-airway epithelium taken by bronchoscopy from cigarette smokers with suspicion of lung cancer [7]. With 16 of the 17 genes available in the dataset, the signature cannot distinguish whether the tumor types of the patients were AD or SCC (data not shown). However, these 16 markers are differentially expressed in the specimens between smokers diagnosed with (N = 59) and without (N = 69) lung cancer (Figure 6). Using their expression as a training set to cross-validate the diagnosis demonstrated 73% sensitivity and 77% specificity, and testing the prediction on an independent dataset (18 cancer and 17 non-cancer cases) showed similar 78% sensitivity and 65% specificity (Figure 7, Additional files 6 and 7). The predicting power of these 16 markers approaches that of the 80-probe biomarker identified in the original paper publishing the datasets (80 to 83% in sensitivity) but far better than that of bronchoscopy diagnosis by cytopathology alone (44 to 53% in sensitivity) [7]. Combining both cytopathology and expression of the 16 markers of the epithelial cells from the training and the validation sets yielded 90% and 89% sensitivity to detect patients with lung cancers, respectively (Figure 7). Again, this also comes near to the original report's 80-gene marker when combining with cytopathology of the cells (95 and 94%, respectively) [7]. However, because the 80-gene marker cannot predict lung cancer subtypes, and cannot distinguish tumors from their adjacent normal tissues [7], the 17 markers identified here might be proven to be more valuable in future diagnostic tests.

## Discussion

There have been a number of studies to directly profile miRNA expression in lung cancers, and unique groups of miRNAs were identified to either characterize the neoplastic tissues or mark patients with poor prognosis [41-43]. This study followed a different rationale to start with three miRNAs enriched in lung but reduced in lung cancers. Since genes targeted by miRNAs determine the final biological activities of these miRNAs, miR-34b/34c/449 is likely to regulate expression of lung cancer markers that might define certain phenotypes of this tumor. Such a rationale transcends the search of predicted target genes with reversed correlation in expression following a miRNA expression profiling, in which any target genes regulated by miRNAs at their protein, not mRNA, levels would have been missed otherwise. Computational predictions of miRNA target genes, followed by a series of data filtering using representation of GO terms and classification of lung cancer specimens, point to a minimal set of 17 genes derived from 251 developmental process genes that correctly predict most of AD and SCC tumors selected from multiple independent cohorts. This gene signature probably represents the least but definitely not the most of miR-34b/34c/449-target genes that participate in tumorigenesis of lung cancer, but the data have suggested the significance of the TGF-beta pathway in these two lung cancer subtypes for future validation and certainly deserves further investigation.

Although miR-34a was initially identified having tumor-suppressing activity together with miR-34b/34c [20], miR-34a is rather ubiquitously expressed in most human tissues and not enriched in lung [9]; yet, this does not exclude the possibility of miR-34a involving in lung cancer tumorigenesis. It might be important to appreciate the fact that miR-34b/34c are expressed in several organs (such as ovary) in additional to lung (unpublished data) in mice, and that whether roles of these two miRNAs underlying lung AD and SCC would be different between human and rodents, since animal models might be used in the future to evaluate the activities of miR-34b/34c/449. It is imperative to recognize that (1) expression of miR-34b/34c/449 in normal tissues is lung-enriched and is reduced in lung cancer in general, whereas many other non-lung-enriched miRNAs could be also greatly differentially expressed between normal and cancerous lung tissues, (2) the predicted miR-34b/34c/449 targets from the developmental process GO term classify AD and SCC better than the predicted targets from other tested GO categories, whereas many other miRNAs and their target genes could be also differentially expressed between AD and SCC, and (3) the 17-gene signature is the final chosen predictor based on their best SAM false detection rate, whereas all 153 genes (the number of genes with eligible data points in Database 1 only) also gave the same prediction error rate as the 17 core genes did. Therefore, it does not preclude the roles of other miR-34b/34c/449 targets, and other miRNAs and their targets. Accordingly, randomized controls, instead of permutation of all available miRNAs/target genes, were taken to ensure the quality of the data, for example, 17 genes were randomly selected from GO categories other than the developmental process term but not from inside the developmental process category.

AD is a collection of heterogeneous tumors with a variety of histologies. For example, as reported previously, AD specimens from the Databases 1, 2, and 3 containing higher percentage of poorly differentiated tumor cells tend to have similar molecular characteristics to SCC and LCLC (namely "squamoid" and "magnoid", respectively), while tumors with a more differentiated phenotype resemble bronchioalveolar carcinoma ("bronchioid") [4]. Staging of the tumors in all the databases analyzed in this study also varies significantly (Table 1). However, expression of the 17 core genes does not seem to associate with these factors, suggesting that this gene expression signature might be independent of histologic features of AD and SCC, degree of differentiation, and progression of disease, and might represent fundamental biologies of the subtypes, such as cellular origins.

This paper embarks a genome-scale meta-analysis strategy that has at least three advantages compared to either a direct profiling of miRNAs in lung cancers or a direct identifying differentially expressed mRNAs in AD and SCC. First, this strategy provides more insights on mechanisms, applications, and functions in connection to the histopathological characteristics underlying AD and SCC of lung. The gene expression signature was identified based on their being predicted targets of three lung-enriched miRNAs, opening the possibility that their expression *in vivo *might be subject to regulation by these miRNAs, and that delivery of these miRNAs might be able to modulate tumorigenic phenotypes, such as TGF-beta pathways that is over-represented in the identified gene signature. Secondly, identifying differentially expressed genes often follows data filtering, which only hones in the most variable expression between the groups, and apparently will discount genes whose differential expression is under the threshold but might be better candidate markers. In fact, several genes in the signature were also identified as differentially expressed between AD and SCC previously (for example, genes that are marked with § in Table 2). Finally, this study avoids identifying predicted target genes whose expression patterns are opposite to those of the miRNAs, but rather depends on their ability to classify the tumor subtypes of interest, especially when no such collection of datasets for miRNA expression available in short term comparable to the size of mRNA expression cohorts used in this study.

Identification of the potential significance of targeting TGF-beta pathways in AD and SCC mirrors previous reports on roles of reduced TGF-beta signaling in lung cancer tumorigenesis [26,27]. Another TGF-beta inhibitory molecule SMAD7 [40] in the 16 TGF-beta pathway genes from the 251 developmental process genes has increased expression in AD than in SCC in all but one datasets (see Additional file 2), which supports the notion that distinctive mechanisms might be used by AD and SCC to suppress TGF-beta pathways.

Despite their differential expression among AD, SCC, and normal lung tissues, the 17-gene signature has no difference in expression in the airway epithelial cells taken from smokers diagnosed with AD or SCC, and most genes had reduced expression in the bronchoscopic specimens from patients with lung cancer, with a few exceptions such as FOS gene (Figure 6). Consistent with the clustering patterns of the Database 1 using this signature in which SCLC was well separate from the other three subtypes (Figure 2D), 6 of 16 cancer cases in the training set predicted as non-cancer are SCLC, compared to only 5 SCLC in 44 predicted positive cases (p = 0.03 by Fisher's exact test). Apparent lower specificity of prediction by this signature in the validation set parallels an interesting pattern of probability distribution in the training set, in which more recent cases seem to have higher probability of being predicted as cancer, suggesting that the follow-up time might not be long enough (see Additional file 2 and its Figure 8 for further discussion). The gene signature identified here can distinguish lung cancer cells from normal lung as well as their peripheral normal tissues (Figure 2D), whereas the markers identified in the article that published the bronchoscopy specimens datasets cannot (although it appeared that the 80-gene signature in the original report can distinguish tumors from normal lung from separate individuals) [7]. It warrants future investigation of a possible two-step diagnostic procedure for cigarette smokers: large-airway epithelial cells obtained by bronchoscopy are examined by both cytopathology and expression of the 17-gene signature, if the subject is predicted as positive and the cancer diagnosis is further confirmed, followed by examining the expression of the same signature in tumor tissues for classification as AD or SCC. Due to the strong association of cigarette smoking with lung cancer and significant false-positive/false-negative rates of lung cancer detection by computed tomography, the findings may significantly impact both basic research and clinical management of lung cancer.

## Conclusion

This study followed an *in silico *strategy to first compile a largest possible list of miRNA targets predicted by multiple algorithms, which was subsequently filtered by GO terms and their enriched representation, and their ability to classify different subtypes of lung cancer. This led to the focus on a list of genes that belongs to the developmental process GO term. Selecting from a final 17-gene expression signature that has the best false detection rate correctly predicts the majority of the AD and SCC subtypes of lung cancer when compared to the histological diagnosis. Most importantly, expression of the same signature in bronchial epithelial cells from cigarette smokers can differentiate whether the patients have lung cancer or not when combined with the cytopathology of the bronchial cells. The results not only provide a functional basis for regulating expression of the markers, but also suggest a two-step diagnostic procedure to smokers: the first step is to combine cytology and expression of the 17-gene signature in large-airway epithelial cells by bronchoscopy; if the subject is predicted as positive and the cancer diagnosis is confirmed, the second step is to classify the tumor as AD or SCC by examining the expression of the same signature in resected or biopsied tumor tissues.

## Abbreviations

AD: adenocarcinoma; SCC: squamous cell carcinoma; NSCLC: non-small cell lung carcinoma; SCLC: small-cell lung carcinoma; LCLC: large-cell lung carcinoma; TGF: transforming growth factor; GO: gene ontology.

## Competing interests

Financial competing interests: The author has been a full-time employee of Applied Biosystems for 3 years and owns common stocks of the company. The processing charge for this article was paid by Applied Biosystems.

Non-financial competing interests: The author declares no non-financial competing interests.

## Pre-publication history

The pre-publication history for this paper can be accessed here:



## Supplementary Material

Additional file 1 **A summary of database selection and the key findings of this study**. Click here for file

Additional file 2 **Extended investigation that was not described in the text due to the interest of space and flow**. Click here for file

Additional file 3 **A complete list of target genes for miR-34b/34c/449 predicted by miRanda, TargetScan, and PicTar**. Click here for file

Additional file 4 **Representation of categories within the Biological Process ontology term (p < 0.05)**. Click here for file

Additional file 5 **Summary of gene lists generated from random controls (predicted targets of miR-141/146b/216 and first and second sets of 17 random genes) and 11 TGF-beta pathway genes from predicted targets of miR-34b/34c/449**. Click here for file

Additional file 6 **Diagnosis and prediction of the bronchoscopic specimens from the training set**. Click here for file

Additional file 7 **Diagnosis and prediction of the bronchoscopic specimens from the validation set**. Click here for file
