# Supplementary material for: An expression meta-analysis of predicted microRNA targets identifies a diagnostic signature for lung cancer
Source: BMC Med Genomics. 2008 Dec 16;1:61. doi: 10.1186/1755-8794-1-61 (PMC2633354; doi:10.1186/1755-8794-1-61)
Supplement: Additional file 2 — Extended investigation that was not described in the text due to the interest of space and flow. [file 1755-8794-1-61-S2.pdf]

A p53-binding site is predicted at the upstream region of the miR-449 gene.

MiR-34b and 34c, together with miR-34a, have been identified as p53-induced tumor suppressor genes [1]. The promoter regions of these three microRNA genes have potential p53-binding sites and have been experimentally verified [2]. Although miR-449 lacks such information as transcriptional regulation and functional roles, a web-based tool [3] that identifies potential cis-regulatory elements by comparative genomics recognizes a p53-binding site within a region about 1.5 kb upstream to the miR-449 gene.

In our previous report we have established a systematic approach to identify potential *cis*-elements responsible for binding of tissue-specific factors for miRNA genes [4]. The miR-449 in our original assays corresponds to the miR-449a gene in current Sanger Database version 10, and a CpG island is identified by UCSC Genome Browser about 2 kb upstream to the miR-449a/449b genes (green bar in Figure 1A). Comparative analysis of the same genomic region between human and mouse sequences revealed that a segment about 1.5 kb upstream to the genes has the highest “Hits” and “Percent Identical” (red circle in Figure 1B), so the sequence of that region was subject to further analysis of transcription factor-binding sites, which showed a p53-binding site at the end of the segment (red circle in Figure 1C). This analysis predicts a putative p53-binding site for miR-449 (both miR-449a and miR-449b) and provides a lead for future investigation on whether miR-449 functions in collaboration with miR-34b and 34c in a p53-regulated fashion.

An important notion is that, although miR-34a was identified together with miR-34b and miR-34c, its expression patterns in human normal tissues are ubiquitous and

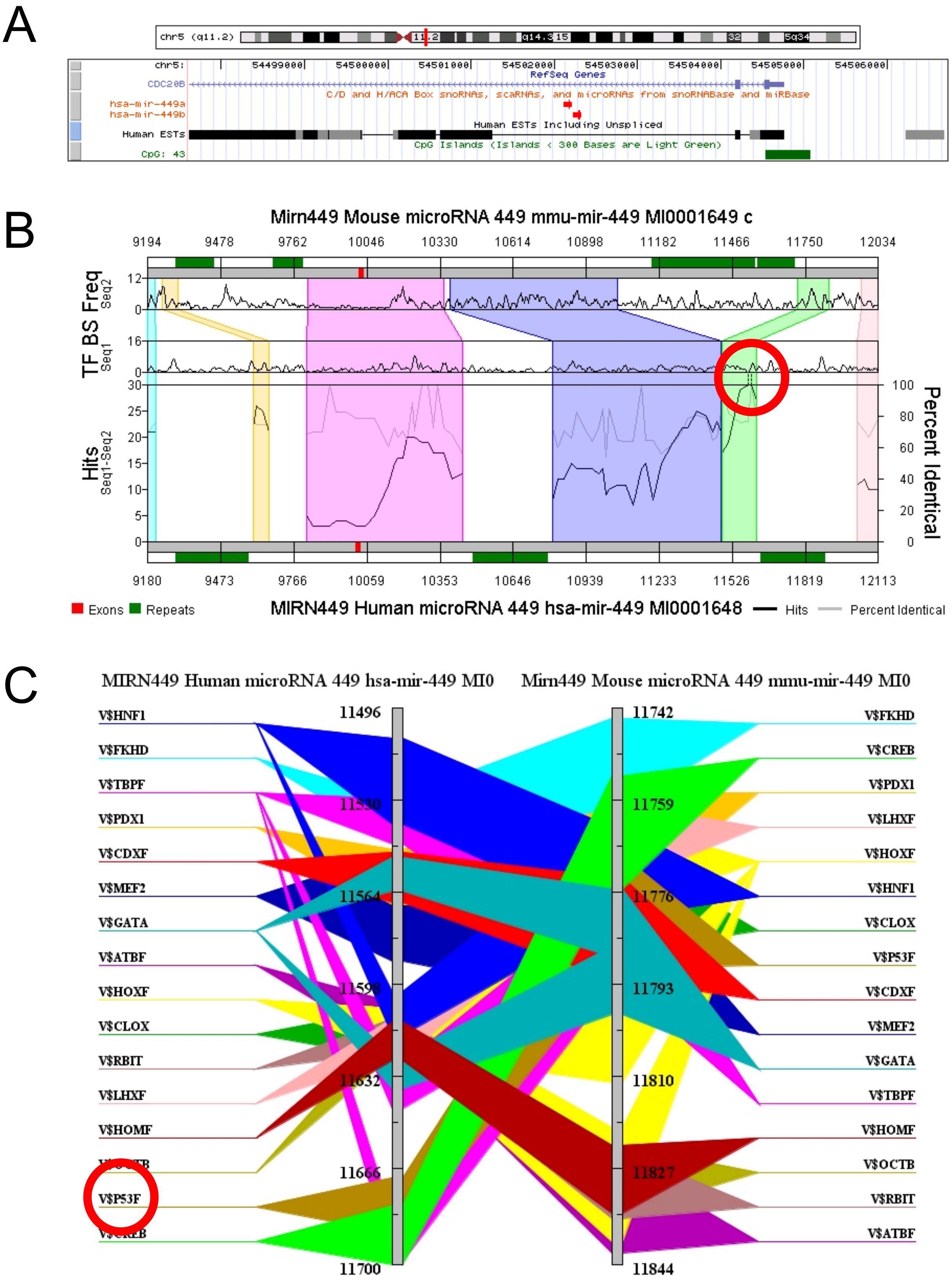

Figure 1. A p53-binding site within a region about 1.5 kb upstream to the miR-449 gene was identified through an in silico strategy.

different from those of miR-34b/34c. Therefore, the meta-analysis of expression of miRNA-predicted target genes in lung cancer only focused on miR-34b and miR-34c that are enriched in lung/trachea, and did not apply to miR-34a.

Expression patterns of miR-34b/34c in NCI-60 cancer cell lines provided hints of the expression characteristics of these three miRNAs in primary lung cancers.

There are four sequences (miR-34b/34bN and miR-34c/34cN) used in our previous published datasets [5]. Mir-34bN and 34cN represent the end sequence variants for miR-34b and miR-34c, respectively. Comparison of the expression levels of miR-34b/34c in 9 lung cancer cell lines among the NCI-60 panel showed that these two miRNAs had higher expression in 2 LCLC cell lines, NCI-H460 and HOP92, than the other 7 NSCLC lines (1 SCC and 6 AD) at statistical significance (all p values less than 0.002, Table 1). These two groups of lung cancer cell lines were similarly separated when a distance matrix of miR-34b/34c expression was used to analyze all cell lines in the NCI-60 panel (all NSCLC cell lines are in the blue bracket as seen in the Figure 3 of the main text), suggesting that expression of miR-34b/34c/449 might be candidates to classify different types of lung cancer. These results are also concordant with a previous finding on expression of miR-34b in NSCLC and LCLC [6]. The calculation of the distance matrix was based on the ΔC_T (the average C_T of miR-34b/34bN/34c/34cN between two cell lines), and hence the smaller the ΔC_T, the more similar expression levels of the four miRNA sequences are in the two cell lines (more red in the heat map).

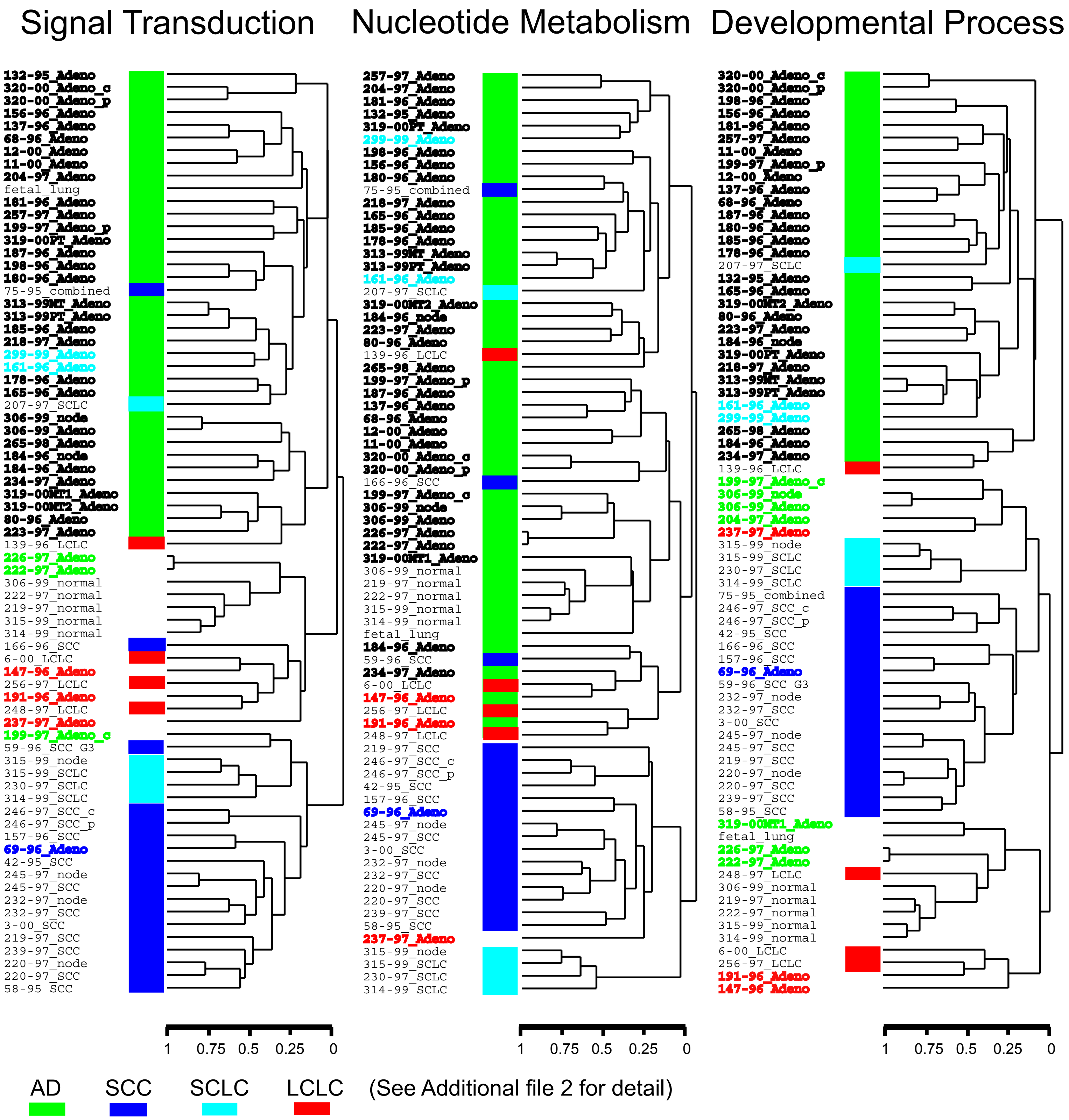
Signal Transduction
132-95_Adeno
320-00_Adeno_c
320-00_Adeno_p
156-96_Adeno
137-96_Adeno
68-96_Adeno
12-00_Adeno
11-00_Adeno
204-97_Adeno
fetal_lung
181-96_Adeno
257-97_Adeno
199-97_Adeno_p
319-00PT_Adeno
187-96_Adeno
198-96_Adeno
180-96_Adeno
75-95_combined
313-99MT_Adeno
313-99PT_Adeno
185-96_Adeno
218-97_Adeno
299-99_Adeno
161-96_Adeno
178-96_Adeno
165-96_Adeno
207-97_SCLC
306-99_node
306-99_Adeno
265-98_Adeno
184-96_node
184-96_Adeno
234-97_Adeno
319-00MT1_Adeno
319-00MT2_Adeno
80-96_Adeno
223-97_Adeno
139-96_LCLC
226-97_Adeno
222-97_Adeno
306-99_normal
222-97_normal
219-97_normal
315-99_normal
314-99_normal
166-96_SCC
6-00_LCLC
147-96_Adeno
256-97_LCLC
191-96_Adeno
248-97_LCLC
237-97_Adeno
199-97_Adeno_c
59-96_SCC G3
315-99_node
315-99_SCLC
230-97_SCLC
314-99_SCLC
246-97_SCC_c
246-97_SCC_p
157-96_SCC
69-96_Adeno
42-95_SCC
245-97_node
245-97_SCC
232-97_node
232-97_SCC
3-00_SCC
219-97_SCC
239-97_SCC
220-97_node
220-97_SCC
58-95_SCC
1 0.75 0.5 0.25 0
Nucleotide Metabolism
257-97_Adeno
204-97_Adeno
181-96_Adeno
132-95_Adeno
319-00PT_Adeno
299-99_Adeno
198-96_Adeno
156-96_Adeno
180-96_Adeno
75-95_combined
218-97_Adeno
165-96_Adeno
185-96_Adeno
178-96_Adeno
313-99MT_Adeno
313-99PT_Adeno
161-96_Adeno
207-97_SCLC
319-00MT2_Adeno
184-96_node
223-97_Adeno
80-96_Adeno
139-96_LCLC
265-98_Adeno
199-97_Adeno_p
187-96_Adeno
137-96_Adeno
68-96_Adeno
12-00_Adeno
11-00_Adeno
320-00_Adeno_c
320-00_Adeno_p
166-96_SCC
199-97_Adeno_c
306-99_node
306-99_Adeno
226-97_Adeno
222-97_Adeno
319-00MT1_Adeno
306-99_normal
219-97_normal
222-97_normal
315-99_normal
314-99_normal
fetal_lung
184-96_Adeno
59-96_SCC
234-97_Adeno
6-00_LCLC
147-96_Adeno
256-97_LCLC
191-96_Adeno
248-97_LCLC
219-97_SCC
246-97_SCC_c
246-97_SCC_p
42-95_SCC
157-96_SCC
69-96_Adeno
245-97_node
245-97_SCC
3-00_SCC
232-97_node
232-97_SCC
220-97_node
220-97_SCC
239-97_SCC
58-95_SCC
237-97_Adeno
315-99_node
315-99_SCLC
230-97_SCLC
314-99_SCLC
1 0.75 0.5 0.25 0
Developmental Process
320-00_Adeno_c
320-00_Adeno_p
198-96_Adeno
156-96_Adeno
181-96_Adeno
257-97_Adeno
11-00_Adeno
199-97_Adeno_p
12-00_Adeno
137-96_Adeno
68-96_Adeno
187-96_Adeno
180-96_Adeno
185-96_Adeno
178-96_Adeno
207-97_SCLC
132-95_Adeno
165-96_Adeno
319-00MT2_Adeno
80-96_Adeno
223-97_Adeno
184-96_node
319-00PT_Adeno
218-97_Adeno
313-99MT_Adeno
313-99PT_Adeno
161-96_Adeno
299-99_Adeno
265-98_Adeno
184-96_Adeno
234-97_Adeno
139-96_LCLC
199-97_Adeno_c
306-99_node
306-99_Adeno
204-97_Adeno
237-97_Adeno
315-99_node
315-99_SCLC
230-97_SCLC
314-99_SCLC
75-95_combined
246-97_SCC_c
246-97_SCC_p
42-95_SCC
166-96_SCC
157-96_SCC
69-96_Adeno
59-96_SCC G3
232-97_node
232-97_SCC
3-00_SCC
245-97_node
245-97_SCC
219-97_SCC
220-97_node
220-97_SCC
239-97_SCC
58-95_SCC
319-00MT1_Adeno
fetal_lung
226-97_Adeno
222-97_Adeno
248-97_LCLC
306-99_normal
219-97_normal
222-97_normal
315-99_normal
314-99_normal
6-00_LCLC
256-97_LCLC
191-96_Adeno
147-96_Adeno
1 0.75 0.5 0.25 0
AD
SCC
SCLC
LCLC
(See Additional file 2 for detail)

Figure 2

Additional file 2

Figure 2. Specimens from the Database 1 (Stanford dataset) were classified using the 251 developmental process genes, as compared to using the signal transduction and nucleotide metabolism genes. A scale bar under each clustering dendrogram represents the correlation coefficients of the nodes of the dendrogram, so the grouping of different lung cancer subtypes can be compared when using different ontology categories for clustering. The color bars between the dendrogram and the sample ID represent the clusters that contain most of the specimens from each subtype, so how well (scattered or concentrated) the samples from each subtype are clustered can be easily visualized. See the Additional file 2 for the colors of the sample ID itself relating to the subtypes.

Table 1. Expression of miR-34b/34c in NCI-60 cancer cell lines.

ΔC_T to normalized expression in normal	NSCLC							LCLC		t test*
	EKVX	A549	NCI-H522	NCI-H226	NCI-H23	NCI-H322M	HOP62	NCI-H460	HOP92	
hsa-miR-34b	8.6	9.4	7.8	9.8	8.9	9.5	9.0	7.0	6.7	0.0016
hsa-miR-34bN	9.0	9.8	8.2	9.3	9.5	9.9	9.0	7.4	7.8	0.0039
hsa-miR-34c	9.5	9.5	8.7	10.2	9.6	10.4	9.2	7.9	7.6	0.0019
hsa-miR-34cN	9.4	10.2	8.6	9.2	9.0	10.3	8.8	7.8	6.5	0.0030

* t tests were used to compare NSCLC and LCLC.

ΔC_T, cancer cell line – normal lung

Tumor subtype-specific clustering of the specimens by the 251 developmental process genes is better than genes from the other 2 categories.

According to the original report that published the Stanford database, AD is divided into 3 subgroups. One of the AD subgroups is together with SCC, SCLC, and LCLC. Each of these 3 subtypes roughly forms their own clusters. Genes from each of the three categories (signal transduction, nucleoside, nucleotide, and nucleic acid metabolism, and developmental processes) were used to classify the same samples by hierarchical clustering (centered gene but not centered sample, and average linkage for clustering), and compare with the dendrogram classification patterns of samples reported by the original authors. As seen in the Figure 2, genes with the nucleotide metabolism ontology term gave the worst sample classification, because AD (green bars) and SCC (dark blue bars) specimens were separate into different clusters. In contrast, signal transduction genes and developmental process genes perform similarly well on classifying AD and SCC. There were several AD specimens that are clustered with other tumor subtypes in the original report. In the Figure 2 they are labeled with the same color as the subtype with which they were clustered together in the original report, for example, light blue for SCLC, red for LCLC, dark blue for SCC, and black for the original AD cluster. The AD with black letters in the main AD cluster was also clustered together in the original report. Seven AD (green letters) that were clustered with most other AD now is scattered to other branches (worse than the original). Two AD (light blue letters) that were clustered with SCLC now is together with most AD (better than the original). Comparing the classification patterns from both signal transduction and developmental process genes, apparently genes from the latter category gave better

separation of subtypes in which all SCC and most AD and SCLC are consolidated in separate clusters (2 SCC specimens are separate from the main SCC cluster in the profiles by signal transduction genes). Collectively, this analysis shows that genes from the developmental process ontology term are better than the other two categories to classify the lung cancer subtypes, especially AD, SCC, and SCLC (see below for more detail).

Predicted target genes of miR-34b/34c/449 ontologically termed with developmental processes were selected to distinguish lung adenocarcinomas from squamous cell carcinomas.

The next step is to evaluate the classification of these 3 lung cancer subtypes by these 251 genes and to validate whether they can correctly predict the same subtypes in the Stanford lung cancer dataset. As shown in the Japanese dataset (Database 6) in Figure 3A, 1 (black circle), 2 (red circle), 3 (green circle), and 4 (blue circle) represent AD, LCLC, SCLC, and COID (carcinoid, only present in this database but not other databases, so it will not be further discussed), respectively. Cross-validation of this dataset using Prediction Analysis of Microarrays (PAM) showed that SCLC has the best separation from the rest of subtypes, followed by AD with moderate accuracy, but almost all LCLC cannot be distinguished. The test prediction of the Stanford dataset (Database 1) in Figure 3B basically follows the same trend, and, most importantly, almost all SCC (“Unspecified” in Figure 3B since there was no SCC in the Database 6) are predicted as SCLC, suggesting that these 251 genes cannot distinguish the SCC

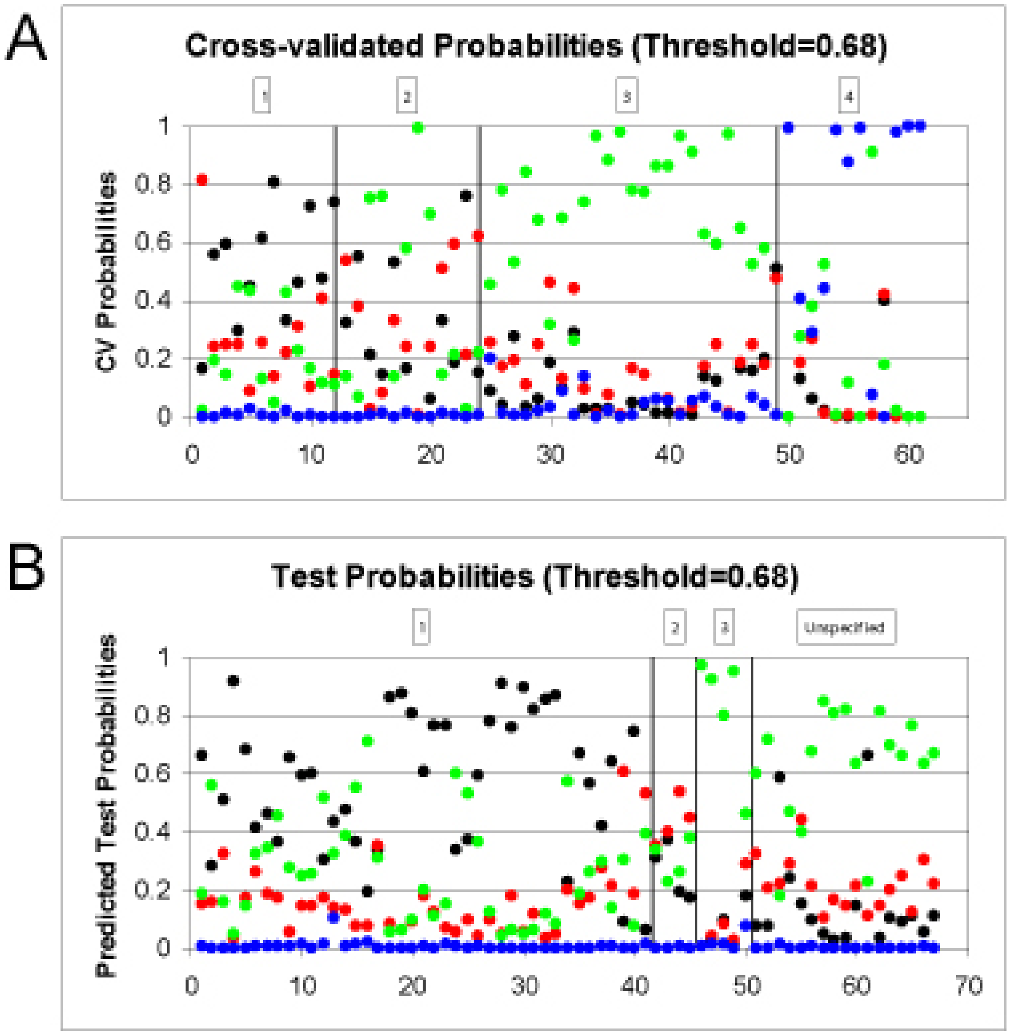

Figure 3. The 251 developmental process genes classify AD and SCC better than the other subtypes.

from SCLC well. Based on these results, these 251 developmental process genes were further evaluated to classify AD and SCC.

Microarray data were adjusted for the Prediction Analysis of Microarrays depending on their platforms and data formats.

In order to perform meta-analysis on multiple databases in which different microarray platforms were used and data were presented in different formats, the microarray data were adjusted to similar distribution patterns based on several guidelines. First, for microarrays spotted with cDNA clones presented with log2-transformed data, such as the Databases 1 and 6, no adjustment was made, and the Database 1 was served as the standard of data adjustment for the rest of datasets. Second, for data from oligonucleotide-arrays (for example, Affymetrix) that has not been log2-transformed, such as the Databases 4, 5, 9, and 12, ratio of each data point to the average expression of the 17 "core" genes across all specimens was calculated, followed by log3-transformation. The reason of using log3 instead of log2 base is because comparing the data distribution showed that log3-transformed data have a better overlap in distribution with data from the Database 1 than log2-transformed counterparts (upper panel of Figure 4). Log2-transformed data in D4 and D5 showed much broader shoulder in distribution. Third, to be consistent on data adjustment, for data from oligonucleotide-arrays that has already been log2-transformed, such as the Databases 7 and 8, each data point was raised to the power of 2 and re-log3-transformed. Another oligonucleotide array Database 13 was log2-transformed but apparently had a distinctive data format from the other nucleotide microarrays, probably

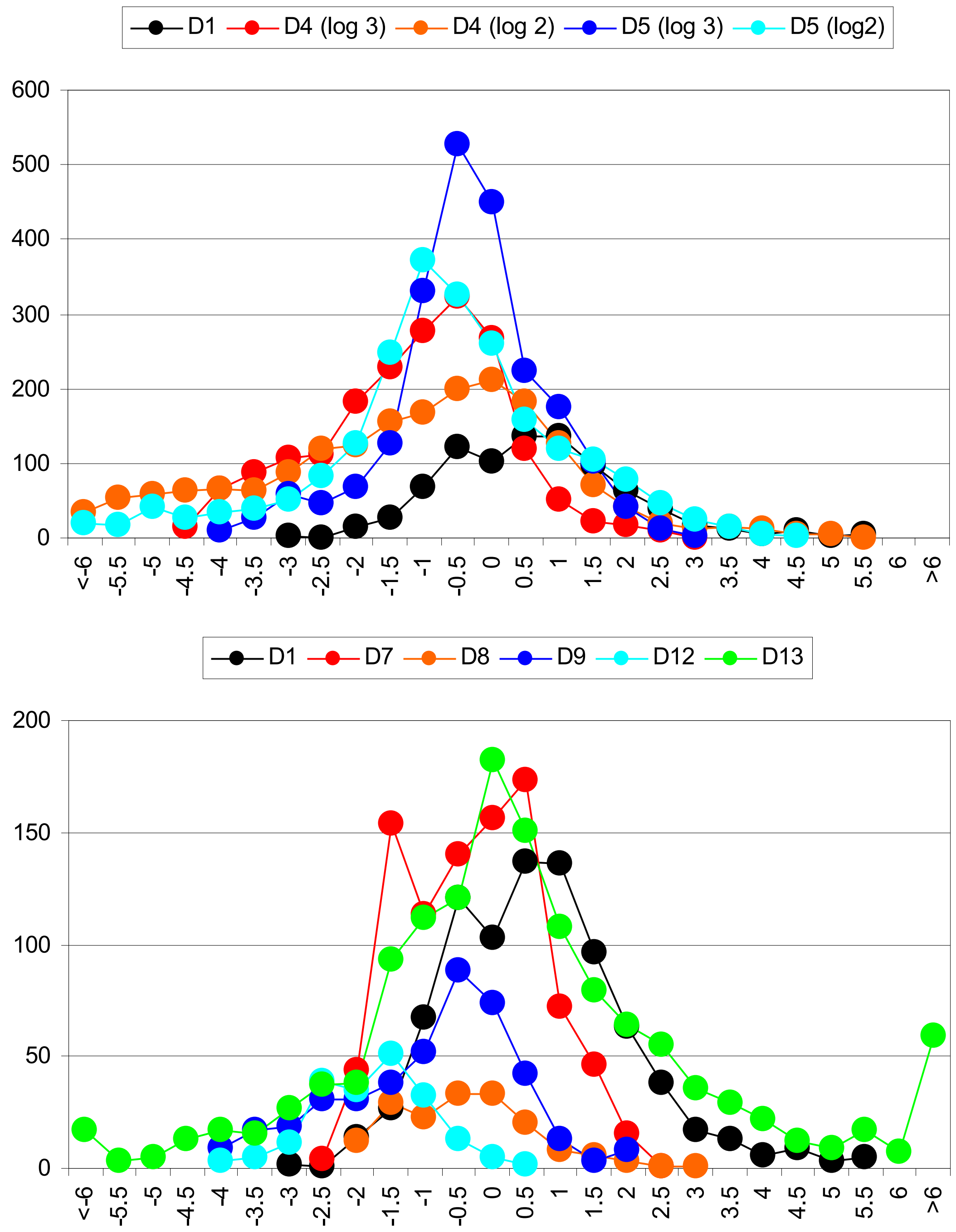

Figure 4. Summary of distribution of data transformed by different log base and power. X-axis, data value; Y-axis, number of data points.

due to its different microarray platform. However, the data can still be adjusted to a similar distribution to the Database 1 by a multiplication of 5 (lower panel of Figure 4). Fourth, for data from the rest of four oligonucleotide-arrays in which their log-base is unknown or data formats are unusual, it was not feasible to use data distribution to adjust those data, so a simple ratio between each data point and the average expression was computed, such as the Databases 10 and 11. Databases 2 and 3 have negative signal values in many data points. Referring to the strategy used by the original article that reported the Database 2 in which all the negative data points were “floored” to 1, the same strategy was also used in the Database 3.

Genes termed with the transforming growth factor-beta signaling pathway are enriched during the course of data filtering.

Overrepresentation of the TGF-beta signaling pathway in the final 17 “core” gene signature does not appear to be a random event, but rather a specific enrichment. Compared to the three TGF-beta pathway genes in the 17 gene signature (Table 2A), this pathway includes 16 genes among the 251 developmental process genes and is the most representative ontology term of all (Table 2B).

Identifying the potential significance of targeting TGF-beta pathways in AD and SCC mirrors previous reports on roles of reduced TGF-beta signaling in lung cancer tumorigenesis [7,8]. Another TGF-beta inhibitory molecule SMAD7 [9] in the 16 TGF-beta pathway genes from the 251 developmental process genes has increased expression in AD than in SCC in all but one datasets. The observation that SCC has up-regulated BMP7 and down-regulated TGFBR2/FOS compared to AD suggests a

Additional file 2

Table 2A. The pathway ontolgoy terms of the 17 core genes with corrected p < 1.

Pathway	NCBI: H. sapiens genes (25431)	Observed (17)	Expected	over/under	p-value (corrected)	Genes
TGF-beta signaling pathway	154	3	0.1	+	0.01980	BMP7, TGFBR2, FOS
Angiogenesis	229	3	0.15	+	0.06320	JAG1, EFNB1, FOS
Unclassified	22565	10	15.08	-	0.22500	

Table 2B. The pathway ontolgoy terms of the 251 developmental process genes with corrected p < 0.05.

Pathway	NCBI: H. sapiens genes (25431)	Observed (251)	Expected	over/under	p-value (corrected)	Genes
TGF-beta signaling pathway	154	16	1.57	+	0.000000001	BMP6, BMP7, CHES1, FOS,FOXA3, FOXG1B, FOXJ2, FOXP1, FOXQ1, FOXR2, MPP2, NODAL, SMAD7, SMOX, ST7, TGFBR2
Angiogenesis	229	18	2.34	+	0.000000007	
Unclassified	22565	198	230.7	-	0.000001230	
Alzheimer disease-presenilin pathway	143	12	1.46	+	0.000005960	
PI3 kinase pathway	121	10	1.24	+	0.000092400	
Insulin/IGF pathway-protein kinase B signaling cascade	94	9	0.96	+	0.000102000	
Notch signaling pathway	51	7	0.52	+	0.000172000	
Interleukin signaling pathway	194	11	1.98	+	0.000932000	
Wnt signaling pathway	349	14	3.57	+	0.002620000	
Cadherin signaling pathway	168	9	1.72	+	0.009890000	

Table 3. Expression of BMP7 and SMAD7 in AD and SCC in 7 datasets.

Database	Database 1		Database 2		Database 4		Database 7		Database 9		Database 10		Database 12	
Subtypes\Ave*	BMP7	SMAD7	BMP7	SMAD7	BMP7**	SMAD7	BMP7	SMAD7	BMP7	SMAD7	BMP7	SMAD7	BMP7	SMAD7
AD	-1.40	0.52	10.74	47.59	385.91	913.40	5.48	5.98	4.79	5.28	4.03	4.34	0.48	0.10
SCC	-0.09	-0.22	41.88	23.65	262.70	746.20	5.86	5.85	5.80	5.02	5.12	4.14	-0.03	-0.14
p value ***	5.8×10^{-5}	2×10^{-4}	0.011	3.6×10^{-6}	0.169	0.023	0.036	0.118	5.5×10^{-6}	0.06	1×10^{-6}	0.038	7.1×10^{-15}	3.8×10^{-5}

* Ave: Average expression level of the gene from all specimens of the same subtype in individual dataset.

** Shaded boxes depict the groups of samples whose expression patterns of BMP7 and SMAD7 are not significantly different, or not coherent with the hypothesis.

general greater activities TGF-beta pathways in AD, but another possibility is that both subtypes have equivalent suppressed TGF-beta activities via distinct mechanisms. The presence of SMAD7 among the 16 TGF-beta pathway genes listed above and its expression patterns are consistent with this notion. Another piece of evidence is that when expression of BMP7 and SMAD7 was collected from 7 datasets that have data available (Table 3), all but one datasets show significant higher expression of SMAD7 in AD than in SCC.

Classification and prediction of AD and SCC subtypes using the miR-34b/34c/449 prediction targets and the 17-gene signature are better than randomized controls.

It is critical to note a theme of this investigation is that identification of the 17-gene signature from predicted targets of miR-34b/34c/449 does not preclude the presence of other miRNAs being differentially expressed between AD and SCC. That said, given the huge number of predicted miRNA targets generated by the union of 3 algorithms, the chance of having some “real” differentially expressed genes hidden in the predicted targets of randomly selected miRNAs might not be small.

Three miRNAs, miR-141/146b/216, were chosen using a random number generator to generate random numbers between 1 and 10,000 for each miRNA in all available assays we used in the published BMC Genomics paper in 2007 and selected three miRNAs with the smallest random numbers. All subsequent procedures followed the same workflow used for the miR-34b/34c/449, including prediction of targets, screening of gene symbols and GO categories/terms, union of all selected genes, and clustering analysis of tumor specimens from the Database 1. Similar to miR-

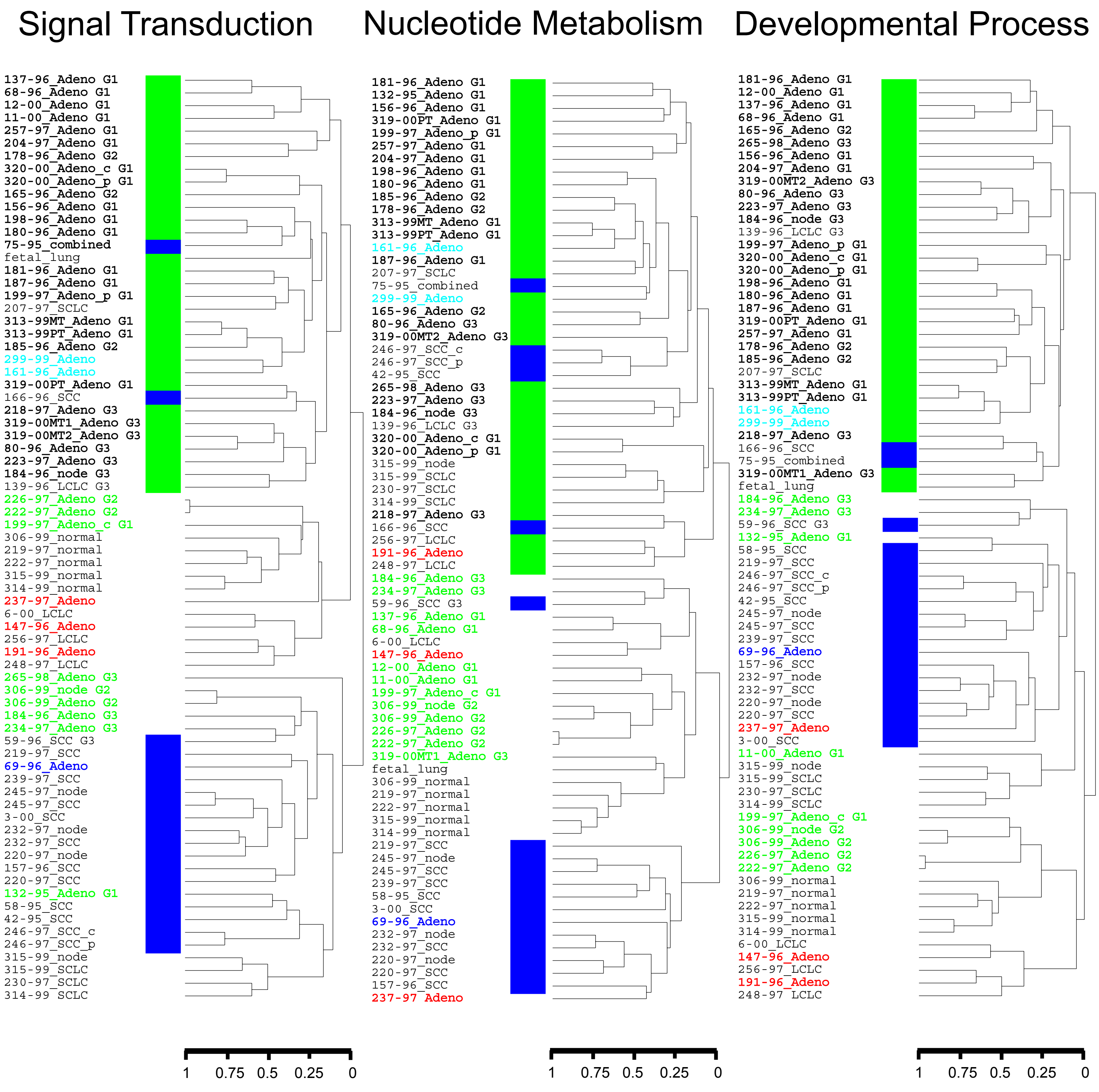

Figure 5. Predicted target genes of randomly selected miR-141/146b/216 that belong to developmental processes, nucleotide metabolism, and signal transduction pathway GO terms were used to hierarchically cluster tumor specimens from the Database 1. Legends are identical to Supplemental Figure 2 except that LCLC and SCLC were not considered in the analysis.

Additional file 2

Table 4. Summary of correlation coefficients (r) of AD/SCC clusters.

	miR-34b/34c/449			miR-141/146b/216		
	Developmental Process	Nucleotide Metabolism	Signal Transduction	Developmental Process	Nucleotide Metabolism	Signal Transduction
# of genes	263	365	368	330	472	469
# of AD outside the main cluster*	6	0	3	8	11	8
r of main AD cluster	0.034	-0.073	-0.015	0.042	-0.017	0.017
r of main SCC cluster	0.154	0.174	0.119	0.119	0.186	0.068
main SCC cluster w other subtypes?**	No	Yes	Yes	Yes	Yes	Yes
# of SCC outside the main cluster	0	3	2	3	6	2
r of cluster including all SCC	0.154	-0.1	-0.12	-0.112	-0.119	-0.126

*This excludes the AD specimens that were already outside the main AD cluster based on the original paper publishing this dataset.

**This excludes the specimens that were clustered w SCC in the original paper publishing the dataset.

34b/34c/449, within the Biological Processes Ontology term, developmental processes, nucleotide metabolism, and signal transduction are the top three categories with most number of genes and lowest corrected p values. The correlation coefficients (r) of the AD and SCC clusters in the original miR-34b/34c/449 analysis and those from the randomized miR-141/146b/216 analysis showed that predicted target genes of miR-34b/34c/449 in the developmental process GO term are clearly better than the others (Figure 5). The comparison of correlation coefficients (r) of AD and SCC clusters between predicted targets of miR-34b/34c/449 and randomly selected miR-141/146b/216 is summarized in the Table 4.

As emphasized above, this investigation does not preclude the roles of other miRNAs. Many miRNAs actually share their target genes at different degree, especially after the union of predicted target genes; these genes will be distilled through the PAM process. Therefore, a better randomized control is a randomly selected list of 17 genes to represent the likelihood of different 17 genes to classify tumor subtypes. Based on this principle, a set of 17 genes from the Database 1 was selected from over 13,000 data points using a random number generator. Cross-validation of PAM showed that the accuracy for AD stays at 93% but for SCC it drops to 65%. Because only a few genes are available in the Database 2, no reasonable prediction could be possibly made. Therefore, a second random list of 17 genes that are available in both Databases 1 and 2 was selected. Although these genes at cross-validation in the Database 1 have 97% and 76% of accuracy for AD and SCC, respectively, they totally failed to identify all SCC specimens in the Database 2. The ability to predict AD and SCC of this second set of random control was further tested on the Databases 4 and 7 that have large number of

both AD and SCC. In the Database 2 the accuracy for AD and SCC is 92% and 45%, respectively, whereas the prediction for the Database 7 totally failed. These results have been summarized in the Figure 6.

A better control for the 3 TGF-beta pathway genes should be other TGF-beta pathway genes among the predicted targets of miR-34b/34c/449 for two reasons. First, this investigation focuses on target genes of these 3 miRNAs; secondly, if the other TGF-beta pathway genes do not classify AD/SCC as well as the results shown in this manuscript, it is the best example to demonstrate that only part, not all, of the TGF-beta pathway genes are responsible for the subtype classification.

Because among the 16 TGF-beta pathway genes from the developmental process GO term, only a few are present in both Databases 1 and 2, the 29 TGF-beta pathway genes from the biological pathway category (including developmental process and other pathways) were used instead (11 out of the 29 are present in both Databases 1 and 2). As sown in the Figure 7, these 11 genes have 88% and 70% of accuracy for AD and SCC, respectively, in cross-validation within the Database 1. Nevertheless, they were completely unable to identify all SCC specimens in the Database 2.

The Additional file 5 summarizes the gene lists from all aforementioned randomized controls.

Expression of the “core” 17-gene signature has the potential to diagnose lung cancer using bronchoscopic specimens taken from cigarette smokers.

The distribution of the cross-validated probabilities of the 69 non-tumor cases predicted by the 16 genes revealed an interesting pattern: cases with larger patient ID

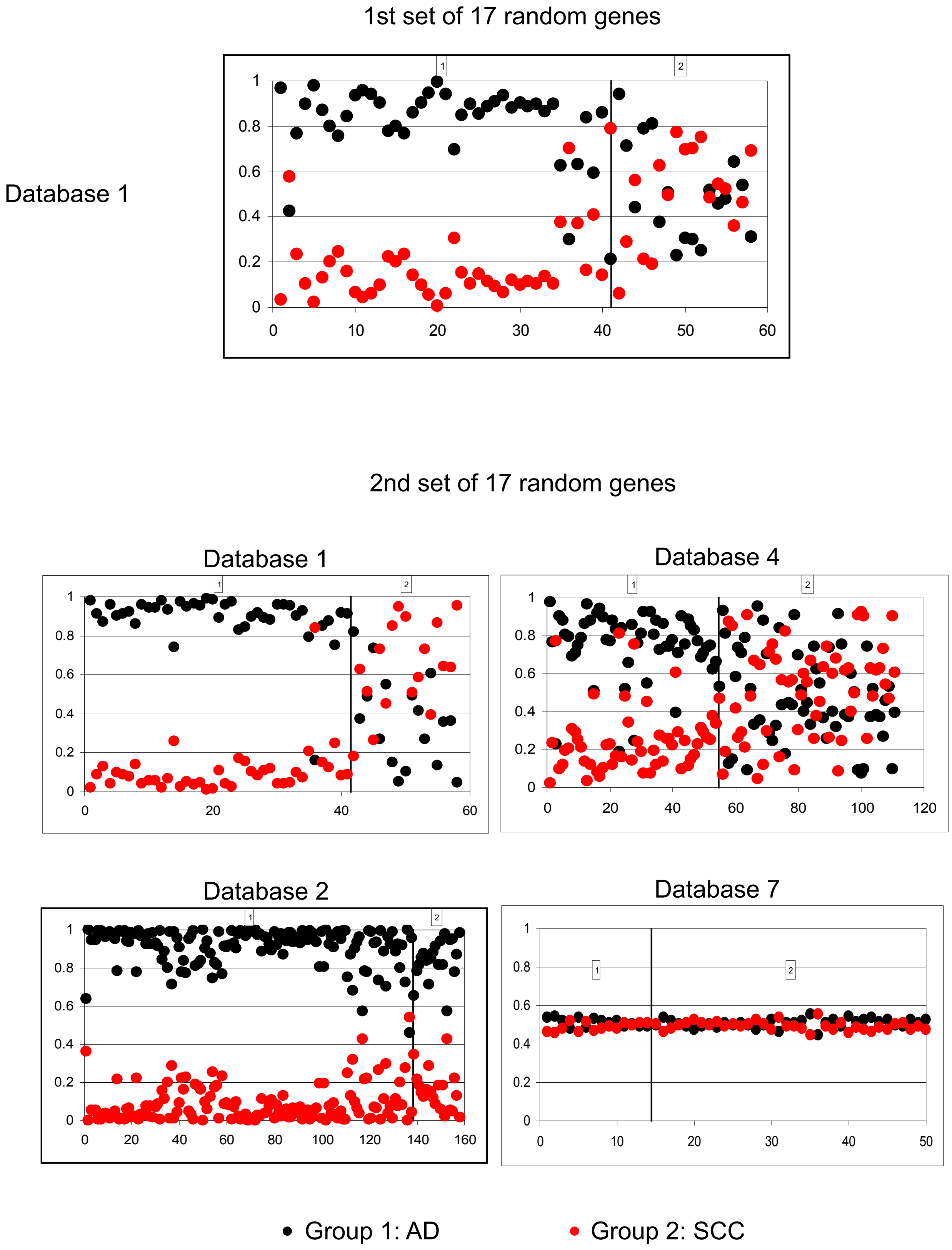

Figure 6. Cross-validation/prediction of AD and SCC in the Databases 1, 2, 4, and 7 using two sets of randomly selected 17 genes. Y-axis, probabilities.

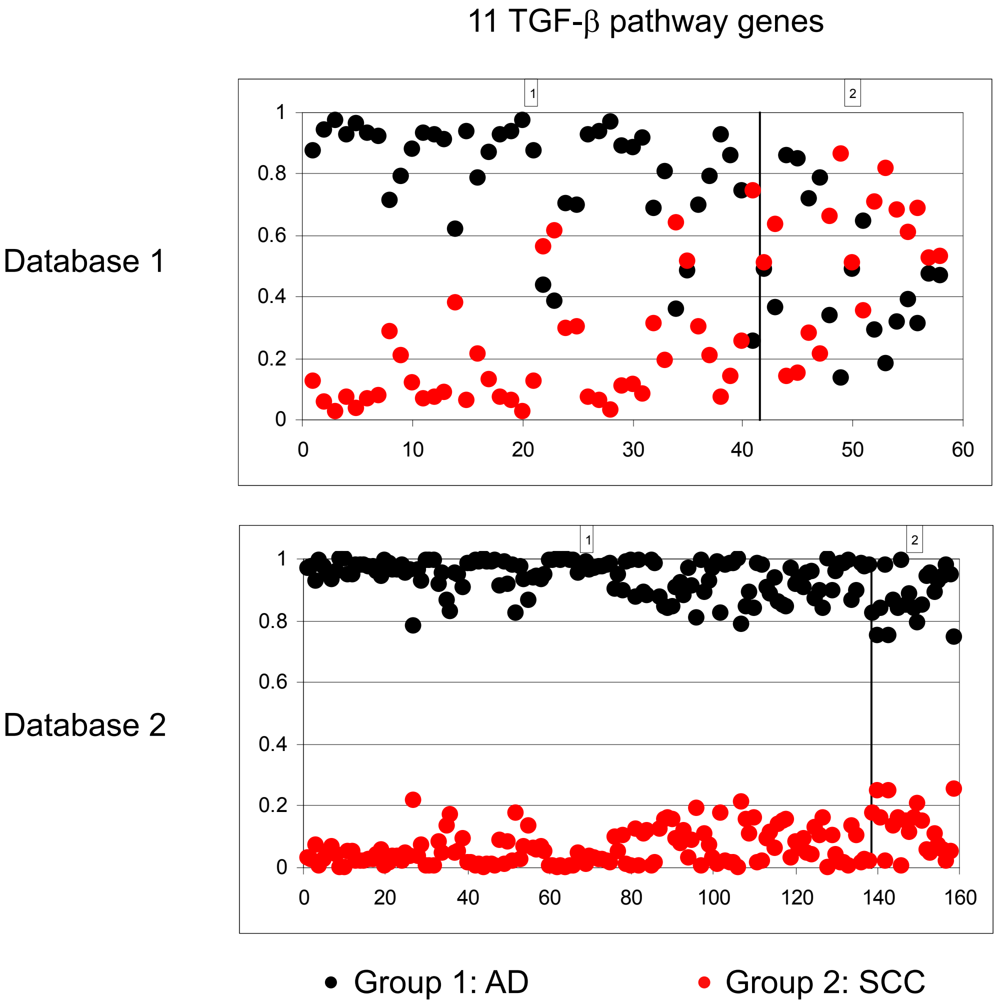

Figure 7. Cross-validation/prediction of AD and SCC in the Databases 1 and 2 using 11 TGF-beta pathway genes derived from the Biological Pathway GO category. Y-axis, probabilities.

numbers, assuming that they are more recent cases, tend to have higher probabilities to be predicted as having tumors (black circles in group 2 of Figure 8). In an attempt to identify clinical correlates associated with this pattern, patients were divided by their median probability (0.1976) of being predicted as having cancer. Among the 69 cases, 54 of them have matched clinical data, with 23 above the median probability and 31 cases below. Fifteen out of the 23 non-tumor cases with above-median probability of being predicted as having tumor had pleural effusions, nodules/mass, squamous metaplasia, fibrosis, infiltrates, and emphysematous changes diagnosed by chest radiographs or bronchoscopy, whereas only 3 of the 31 cases with below-median probability had such clinical presentations (p=0.000026 by Fisher's exact test). This result suggests that some of these cases might eventually develop lung cancer, and that the tumor cells remained elusive at the time of their publication is because not enough time was given for follow-up. It will be of great value to continue to follow up the patients to verify this hypothesis in the future.

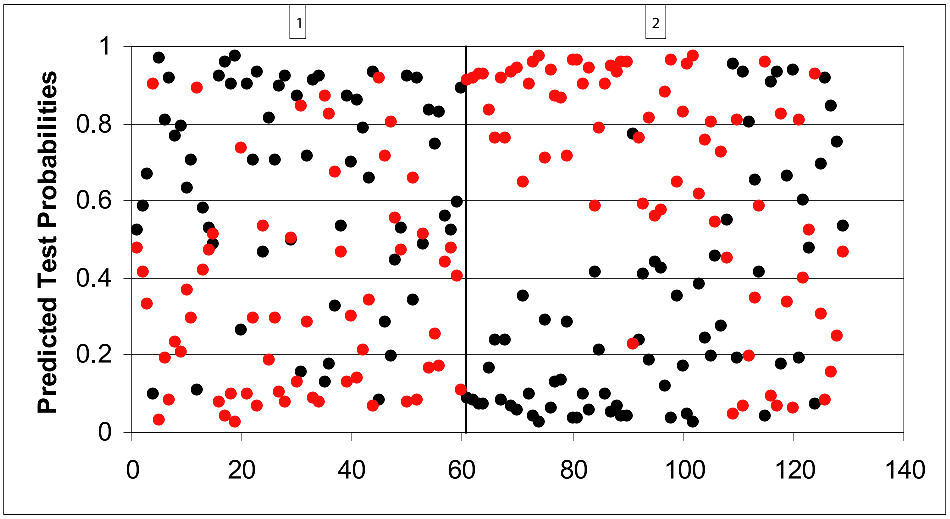

Group 1: Cancer Group 2: Non-cancer

Figure 8. The distribution of the cross-validated probabilities of the 69 non-tumor cases predicted by the 16-gene signature revealed an interesting pattern. Y-axis, probabilities.

References

1. He L, He X, Lowe SW, Hannon GJ (2007) microRNAs join the p53 network--another piece in the tumour-suppression puzzle. Nat Rev Cancer 7: 819-822.

2. He L, He X, Lim LP, de Stanchina E, Xuan Z, et al. (2007) A microRNA component of the p53 tumour suppressor network. Nature 447: 1130-1134.

3. Jegga AG, Chen J, Gowrisankar S, Deshmukh MA, Gudivada R, et al. (2007) GenomeTrafac: a whole genome resource for the detection of transcription factor binding site clusters associated with conventional and microRNA encoding genes conserved between mouse and human gene orthologs. Nucleic Acids Res 35: D116-121.

4. Liang Y, Ridzon D, Wong L, Chen C (2007) Characterization of microRNA expression profiles in normal human tissues. BMC Genomics 8: 166.

5. Gaur A, Jewell DA, Liang Y, Ridzon D, Moore JH, et al. (2007) Characterization of microRNA expression levels and their biological correlates in human cancer cell lines. Cancer Res 67: 2456-2468.

6. Bommer GT, Gerin I, Feng Y, Kaczorowski AJ, Kuick R, et al. (2007) p53-mediated activation of miRNA34 candidate tumor-suppressor genes. Curr Biol 17: 1298-1307.

7. Anumanthan G, Halder SK, Osada H, Takahashi T, Massion PP, et al. (2005) Restoration of TGF-beta signalling reduces tumorigenicity in human lung cancer cells. Br J Cancer 93: 1157-1167.

8. Borczuk AC, Papanikolaou N, Toonkel RL, Sole M, Gorenstein LA, et al. (2007) Lung adenocarcinoma invasion in TGFbetaRII-deficient cells is mediated by CCL5/RANTES. Oncogene.

9. Itoh S, Landstrom M, Hermansson A, Itoh F, Heldin CH, et al. (1998) Transforming growth factor beta1 induces nuclear export of inhibitory Smad7. J Biol Chem 273: 29195-29201.
